# Single-cell RNA sequencing reveals transcriptional changes of human choroidal and retinal pigment epithelium cells during fetal development, in healthy adult and intermediate age-related macular degeneration

**DOI:** 10.1093/hmg/ddad007

**Published:** 2023-01-16

**Authors:** Joseph Collin, Megan S R Hasoon, Darin Zerti, Sarah Hammadi, Birthe Dorgau, Lucy Clarke, David Steel, Rafiqul Hussain, Jonathan Coxhead, Steven Lisgo, Rachel Queen, Majlinda Lako

**Affiliations:** Biosciences Institute, Faculty of Medical Sciences, Newcastle University, Newcastle, NE1 3BZ, UK; Biosciences Institute, Faculty of Medical Sciences, Newcastle University, Newcastle, NE1 3BZ, UK; Biosciences Institute, Faculty of Medical Sciences, Newcastle University, Newcastle, NE1 3BZ, UK; Microscopy Centre and Department of Applied Clinical Sciences and Biotechnology, University of L’Aquila, L'aquila 67100, Italy; Biosciences Institute, Faculty of Medical Sciences, Newcastle University, Newcastle, NE1 3BZ, UK; Biosciences Institute, Faculty of Medical Sciences, Newcastle University, Newcastle, NE1 3BZ, UK; Department of Ophthalmology, Royal Victoria Infirmary and Newcastle University, Newcastle, NE1 4LP, UK; Biosciences Institute, Faculty of Medical Sciences, Newcastle University, Newcastle, NE1 3BZ, UK; Biosciences Institute, Faculty of Medical Sciences, Newcastle University, Newcastle, NE1 3BZ, UK; Biosciences Institute, Faculty of Medical Sciences, Newcastle University, Newcastle, NE1 3BZ, UK; Biosciences Institute, Faculty of Medical Sciences, Newcastle University, Newcastle, NE1 3BZ, UK; Biosciences Institute, Faculty of Medical Sciences, Newcastle University, Newcastle, NE1 3BZ, UK; Biosciences Institute, Faculty of Medical Sciences, Newcastle University, Newcastle, NE1 3BZ, UK

**Keywords:** embryonic and fetal eye, retinal pigment epithelium, choroid, choroid endothelial cells, melanocytes, pericytes, smooth muscle cells, Schwann cells, single cell RNA-Seq, age related macular degeneration (AMD)

## Abstract

Age-related macular degeneration (AMD) is the most prevalent cause of blindness in the developed world. Vision loss in the advanced stages of the disease is caused by atrophy of retinal photoreceptors, overlying retinal pigment epithelium (RPE) and choroidal endothelial cells. The molecular events that underline the development of these cell types from *in utero* to adult as well as the progression to intermediate and advanced stages AMD are not yet fully understood. We performed single-cell RNA-sequencing (RNA-Seq) of human fetal and adult RPE–choroidal tissues, profiling in detail all the cell types and elucidating cell type-specific proliferation, differentiation and immunomodulation events that occur up to midgestation. Our data demonstrate that progression from the fetal to adult state is characterized by an increase in expression of genes involved in the oxidative stress response and detoxification from heavy metals, suggesting a better defence against oxidative stress in the adult RPE–choroid tissue. Single-cell comparative transcriptional analysis between a patient with intermediate AMD and an unaffected subject revealed a reduction in the number of RPE cells and melanocytes in the macular region of the AMD patient. Together these findings may suggest a macular loss of RPE cells and melanocytes in the AMD patients, but given the complex processing of tissues required for single-cell RNA-Seq that is prone to technical artefacts, these findings need to be validated by additional techniques in a larger number of AMD patients and controls.

## Introduction

Age-related macular degeneration (AMD) is a progressive degenerative disease of the macular region of the retina, leading to decline and eventual loss of central vision (1). It is the third leading cause of visual loss globally (World Health Organization), with 170 million individuals affected globally and nearly 300 million people predicted to have AMD by 2040 (2), posing a substantial socioeconomic burden. Drusen accumulation and abnormalities in the retinal pigment epithelium (RPE) are key features of AMD. The disease progresses from an early stage characterized by medium sized drusen, through intermediate stages characterized by retinal pigmentary changes, to the late stages of either dry (geographic atrophy) or wet (neovascular) AMD (1). In the advanced stages of the disease, death of retinal photoreceptors, RPE and choroid endothelial cells (CECs) are common.

The choroid is a highly specialized systemic vascular network, lying just beneath the RPE, between Bruch’s membrane and the sclera. It is composed of three layers: the inner most layer (choriocapillaris), middle (Sattler’s) and outer layer (Haller’s layer). The choriocapillaris is a continuous layer of fenestrated capillaries with surrounding pericytes organized in a lobular arrangement and is the principal blood supply to the RPE and photoreceptors. A key characteristic of CECs is the presence of multiple fenestrations, allowing for directional flow of oxygen and nutrients by diffusion from the choroid to the RPE and to the inner segments of photoreceptors (3), and the removal of waste products from the RPE for systemic recycling. Importantly, the choriocapillaris supplies the central retinal region responsible for high acuity vision (fovea), which lacks retinal blood vessels (4). While in the short term this augments the local supply of oxygen and nutrients, in the longer term it makes the fovea more vulnerable to age-related changes, which create barriers to effective diffusion of oxygen and nutrients from the choriocapillaris (5).

The development of the choroid in humans starts from the fourth week post conception (PCW) (6). Fate mapping studies in mice indicate that CECs are derived from mesoderm, whereas the other cell types are derived from the neural crest (6). At 4–6 PCW, haemangioblast cells expressing endothelial (CD31), haematopoietic (CD34) and angioblast markers (CD39, VEGFR2) are observed in island like formations in the choriocapillaris and scattered within the forming choroidal stroma (7). By 6.5 PCW, a single layer of vasculature is present in the choroid, forming the choriocapillaris. The formation of deeper choroidal vessels is observed at 9–10 PCW; however, no functional fenestrations are present. Four weeks later, a few fenestrations are present as well as endothelial cell junctions and a more organized Bruch’s membrane. At 18–19 PCW, three layers of blood vessels are present in the posterior pole region, coinciding with the first evidence of photoreceptor maturation. At this stage of development, the choriocapillaris is composed of thin-walled, flat blood vessels with lumens, well-defined tight junctions and continuous areas of fenestration. Bruch’s membrane is also more developed with interspersed collagen and elastin beneath the RPE basement membrane (7). At 20 PCW choriocapillaris has not yet acquired a lobular pattern, indicating that a significant expansion and remodelling occurs after this time point. Although RPE pigmentation occurs early in human development, choroid pigmentation develops later at approximately the eighth month (8). Data describing choroidal development are scarce, mostly because of paucity of reliable techniques for measuring choroidal thickness *in utero*. Two studies have shown an association between choroidal health and birth weight, body length and gestational age (9,10), demonstrating that children born at lower birth weight had a thinner choroid during adolescence.

As with most tissues, the choroid undergoes various changes during the ageing process, with these being more severe in patients with AMD. One of these changes is reflected in a decrease in choroidal thickness with age, with the choroid underneath the fovea reducing by ~3 μm per year in healthy adults (age 21–85 years old) (11). In wet AMD patients, reduced circulation within the choroid is accompanied by a decrease in choriocapillaris density and an increase in the presence of ghost vessels (12). In patients with dry AMD, altered abundance and/or function of proteases as well as a thinner choroid is observed (13). Membrane attack complex (MAC) deposition in Bruch’s membrane and choriocapillaris has been observed in very young eyes; however, MAC deposition increased with age, with the highest levels observed in eyes with AMD (14). Accumulation of the MAC results in complement mediated CECs lysis that together with elevated monomeric C-reactive protein create an inflammatory environment and promote disease progression (15). Together these findings suggest that understanding the development, and changes that occur during ageing and disease processes, are essential to better evaluate the tissue in normal physiological conditions and disease states, and also provide insight into what types of treatments may be the most useful to stop or reverse AMD or other diseases affecting this tissue.

To date, most gene expression studies of choroid and RPE have used mRNA or protein samples from pooled RPE–choroid as these two tissues are difficult to separate. Furthermore, the cellular diversity within the choroid has prevented assignment of unique gene expression signatures to individual cell types. The advances made in the single-cell gene expression and chromatin accessibility studies have provided a powerful approach to study gene expression patterns in adult RPE and choroid (16,17); however, a full developmental molecular map of human choroid development and overlaying RPE and how these tissues change with age and disease has not been generated to date. Our hypothesis is that the single-cell RNA-Seq data obtained from this study would provide the molecular map of normal development as well as the benchmark for assessing the maturity of CECs and RPE cells generated from human pluripotent stem cells. Importantly, the comparison of single-cell data from RPE–choroid tissues of healthy individuals to these with age-related and inherited retinal disease can provide significant insights into disease pathogenesis and yield disease biomarkers. In this manuscript, we report the single-cell RNA-seq analyses of RPE–choroidal tissues from 12 to 21 PCW and adult post-mortem specimens with particular focus on CECs, melanocytes, smooth muscle cells, pericytes, Schwann cells and RPE. Importantly we compare these cell clusters between a person with intermediate AMD and an unaffected subject using RPE–choroid tissue retrieved from the macular and peripheral regions, drawing important insights for AMD progression.

## Results

### Single-cell RNA-Seq of human fetal RPE–choroid samples

Four fetal RPE–choroid samples were dissected from the retina and the sclera of four eyes of 12, 16, 20 and 21 PCW specimens, dissociated to single cells and profiled by single-cell RNA-Seq (Supplementary Material, Table S1). Samples were profiled to a mean depth of 13 062 counts/cell and 2916 genes/cell. A total of 11 793 cells were recovered from these four fetal samples following quality control and doublet-cell exclusion. Uniform manifold approximation and projection (UMAP) dimensionality reduction, and Seurat graph-based clustering was performed for each sample. Cluster identification was performed using the cell specific markers reported by Voigt *et al.* (16) in adult RPE–choroid samples (Supplementary Material, Figs S1 and Supplementary Material, S2; Supplementary Material, Table S1). Proliferating cells characterized by high expression of *Ki67, TOP2A* and *CDK1* were present in the 12 and 16 PCW samples, but not in the latter 20 and 21 PCW specimens, consistent with the process of terminal differentiation that occurs very early during development for most of the cell types found in the RPE–choroid tissue (18,19). Although we endeavoured to cleanly dissect the RPE–choroid from other eye structures, a small fraction of other cell types including retinal cells such as rod photoreceptors (characterized by expression of *NRL*), bipolar (characterized by expression of *VSX1*) and amacrine cells (characterized by expression of *TFAP2A*), iris pigmented epithelium (characterized by expression of *WNT2B, TFPI2, DCT, PMEL, TYR*), ciliary muscle (characterized by expression of *ACTA2, MYH11, MYL9, WNTB2*), red blood cells (characterized by expression of *HBG, HBA1, HBB*) and corneal epithelium (characterized by expression of *KRT12*) was observed in some of the samples, but not all. Several neural crest-derived cell clusters representing fibroblasts, pericytes and periocular mesenchyme were observed in all samples (Supplementary Material, Figs S1 and Supplementary Material, S2; Supplementary Material, Table S1).

**Figure 1:**
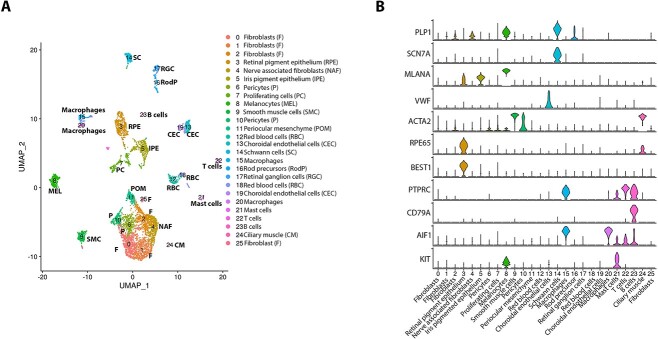
Single-cell RNA-Seq of fetal human RPE–choroid tissue. (**A**) Integrated UMAP of RPE–choroid tissue from 12 to 21 PCW revealing the presence of 26 cell clusters. (**B**) Violin plots showing the expression of key cell type-specific markers.

The reads from the four fetal samples (12, 16, 20, 21 PCW) were integrated using the harmony batch correction (Fig. 1). The cells at different PCWs were downsampled to 1000 cells per sample. As in the separate samples, several clusters with high expression of neural crest markers, *PITX2* and *FOXC1* (20), were identified, with clusters 0, 1 and 2 expressing markers of fibroblasts including *PENK, COL1A1, FBLN1*, clusters 6 and 10 expressing pericyte markers (*THY1, ITGA1, POSTN*), cluster 9 expressing typical smooth muscle cell markers (*ACTA2, TAGLN, DES*), cluster 11 expressing periocular mesenchyme markers (*MGP, DCN*) and cluster 4 expressing at high level nerve associated fibroblast markers [*SFRP4*, *PI16* (21)]. The pigmented cell clusters were easily identified as melanocytes (cluster 8) and RPE (cluster 3) by the high expression of *MLANA* and *RPE65*, respectively (Supplementary Material, Table S1). We were able to identify iris pigment epithelium cluster 5 associated with high expression of the characteristic marker *WNT2B* (22) and pigmentation markers *PMEL* and *TYR* (cluster 5) as well as a ciliary muscle cluster 24, with high expression of *PAX6, WNT2B* and muscle related markers such as *MYL9, MYH11* and *DES*. Immune cells, including T and B cells (clusters 22 and 23), mast cells (cluster 21) and macrophages (clusters 15, 20) were recovered from the tissue, along with Schwann cells (cluster 14) and numerous CECs which formed two-closely related clusters 13 and 19, characterized by high expression of *VWF, ICAM2* and *CD34*. Similar to separate samples, retinal cells including rod photoreceptor precursors (cluster 16) and ganglion cells (cluster 17) were identified in the integrated analysis alongside abundant red blood cells (clusters 12, 18).

**Figure 2:**
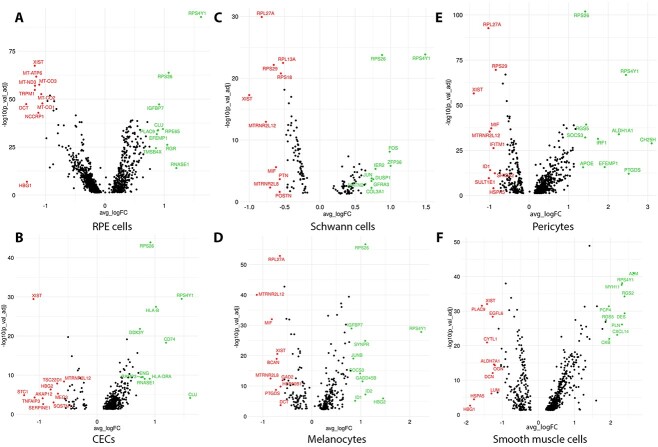
Volcano plots showing differentially expressed genes between early and late fetal development in RPE cells (**A**), CECs (**B**), Schwann cells (**C**), melanocytes (**D**), pericytes (**E**) and smooth muscle cells (**F**). Top 10 upregulated and downregulated genes are shown in green and red colour, respectively. A false discovery rate (FDR) threshold of <0.05 was applied.

Differential expression gene (DEG) analysis was carried out to identify the genes whose expression changes significantly during the development of the RPE–choroid tissue. To this end, genes expressed in the RPE, CECs, pericytes, smooth muscle, Schwann cells and melanocytes were compared between the earlier (12 and 16 PCW) and later (20 and 21 PCW) stages of development of the second trimester (Supplementary Material, Table S2, Fig. 2). Two key genes, namely *RPE65* and *RGR* (23), encoding proteins involved in the visual cycle in a sequence of reactions that recycles 11-cis retinal, were amongst the top 10 most significantly upregulated genes in late fetal stages (Fig. 2A), demonstrating RPE maturation (24), which goes hand to hand with suppression of cell proliferation orchestrated by transforming growth factor β1 (25) (Supplementary Material, Table S3). These changes in gene expression were gradual over the developmental time course studied herein (Supplementary Material, Fig. S3A). RPE cells reside in an oxygen-rich environment and RPE mitochondrial DNA is prone to oxidative stress and damage. Neural retina and RPE are thought to be part of a metabolic ecosystem, where each cell type is dependent on the other for the survival (26). According to this model, glucose from choroidal blood is not used by the RPE cells, but instead is transported to the photoreceptors, where it is used in glycolysis to generate energy. Lactate, a by-product of glycolysis is transported back to RPE cells where it is used for oxidative phosphorylation, which occurs in the inner mitochondrial membrane. Although oxidative phosphorylation is an essential part of metabolism, it generates reactive oxygen species (ROS), resulting in oxidation of proteins, lipids and nucleic acids, ultimately triggering RPE degeneration (27,28). The DEG analysis coupled with enrichment of canonical pathways suggest that the RPE cells from 20 to 21 PCW are able to supress oxidative phosphorylation (Supplementary Material, Fig. S4, Supplementary Material, Table S4) via downregulation of several mitochondrial genes encoding components of the cytochrome oxidase (*MT-CO1, MT-CO2, MT-CO3*) and those involved in complex I (*MT-ND1, MT-ND2, MT-ND4,*  Fig. 2A, Supplementary Material, Fig. S3A, Supplementary Material, Table S2) as well as increase the antioxidant defence through the activation of the sirtuin signalling pathway (Supplementary Material, Fig. S4) (26), thus reducing the impact of damaging ROS during RPE differentiation.

The human choriocapillaris expresses human leukocyte antigen (HLA)-class I self-peptides at high levels (29), however it has not been established at which stage of development the CECs acquire the expression of these peptides. The DEG analysis (Supplementary Material, Table S2, Fig. 2B, Supplementary Material, Fig. S3B) combined with upstream regulator (Supplementary Material, Table S3) and canonical pathway enrichment (Supplementary Material, Table S4) revealed a significant and gradual upregulation of several genes encoding HLA-Class I (*HLA-B* and *HLA-A*), but also some genes encoding HLA-class II proteins (*HLA-DRA* and *HLA-DMA*), suggesting activation of antigen presentation pathway at midgestation. CECs transplantation in AMD patients has not been carried out, however given the expression of both HLA-class I and II peptides on fetal CECs, appropriate strategies must be designed to avoid transplanted cell rejection because of host’s immune response.

It is known that the choroid is richly innervated by parasympathetic, sympathetic and trigeminal sensory nerve fibres that regulate choroidal blood flow in birds and mammals (30). Schwann cells perform a number of functions, key amongst which are the ensheathment and myelination of nerve fibres, promotion of retinal ganglion cell (RGC) axon regeneration (31), production of trophic factors, preservation of retinal tissue through reduction of oxidative stress (32) and restoration of electrophysiological conditions (33). Predicted activation of upstream regulators including key growth factors (epidermal growth factor (EGF), insulin-like growth factor (IGF1)) (34,35), pro-inflammatory signals (TNFα, IL6) (36,37) and inhibition of RB1 regulator, which serves as a brake to stop G1 to S cell phase transition (Supplementary Material, Table S3), suggests that Schwann cells are still in a proliferative state as development proceeds from 12 PCW to midgestation.

Human choroidal melanocytes are responsible for choroidal pigmentation: they absorb light, regulate free radical production and modulate inflammation (38). For a long while it was thought that melanocytes migrate to the choroid in the third trimester during primate development (8,39,40). However, work performed in mice, has shown that melanocytes arise from melanoblasts, which differentiate within the developing neural crest-derived mesenchyme that envelops the optic cup and invade the choroid much earlier in development than previously thought (41). Melanocyte inducing transcription factor (MITF), a master regulator of melanocyte development is expressed at high levels in melanoblasts as they differentiate into melanocytes (42). The high expression of this transcription factor alongside other pigmentation markers such as *PMEL* and *TYRP1*, demonstrates the presence of differentiating melanoblasts as early as 12 PCW of human choroid development (Supplementary Material, Fig. S1). The expression of these markers increases significantly and gradually in the melanocyte clusters from 12 to 21 PCW (Supplementary Material, Table S2, Supplementary Material, Fig. S3C), suggesting a continuous process of terminal differentiation.

Pericytes are a heterogenous population of cells located in the blood vessel wall. While in the retina the ratio of CECs to pericytes is 1:1, in the choroid the ratio of CECs to pericytes is ⁓50% of that observed in the other eye microcirculations (43). Importantly, the retinal pericytes are highly immunosuppressive and they protect CECs from inflammation-mediated apoptosis. It is not known if this immunomodulatory activity is intrinsic to pericytes or acquired during the differentiation process. Our data suggest that the later may be true, for a key upstream regulator, interferon γ (IFNG), which is able to suppress the proliferation of host effector memory cells is activated as the development proceeds from 12 to 21 PCW (Supplementary Material, Table S3).

It has been shown that during development vascular smooth muscle cells migrate to the endothelial tubes, whereas simultaneously undergoing a programme of differentiation. Upon incorporation into the vasculature, vascular smooth muscle cells become quiescent and primarily regulate vascular tone, whereas still retaining the ability to proliferate in response to vascular injury (44). This process of differentiation is orchestrated by activation of RhoA and p38 signalling pathways, which in turn activate MEF2C followed by activation of myocardin that together with the serum response factor, induce smooth muscle marker gene expression (45). Our pathway enrichment (Supplementary Material, Table S4) and upstream regulator analysis (Supplementary Material, Table S3) recapitulate these differentiation steps, demonstrating that the smooth muscle cells are undergoing differentiation during 12–21 PCW of development.

### Single-cell RNA-Seq of human adult RPE–choroid tissue

**Figure 3:**
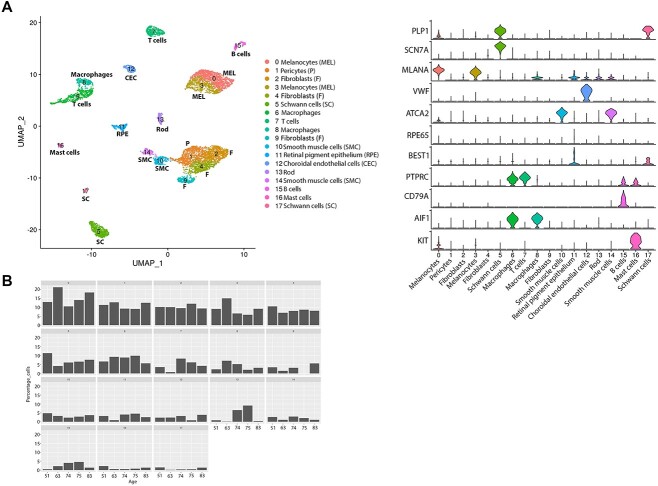
Single-cell RNA-Seq of adult human RPE–choroid tissue. (**A**) Integrated UMAP (left) revealing the presence of 18 cell clusters in the adult RPE–choroid tissue and violin plots (right) showing the expression of key cell type specific markers. (**B**) Cell type representation in RPE–choroid tissue across five different donors of different ages. The age is shown on the *x* axis and cluster number on top of each panel.

The RPE–choroid regions were dissected from five adult donor eyes (age range 51–83 years old, Supplementary Material, Table S1), dissociated to single cells and profiled by single-cell RNA-Seq. Samples were profiled to a mean depth of 10 089 counts/cell and 2400 genes/cell. The samples were integrated by harmony batch correction. The cells from three donors were downsampled to 2000 cells, from one donor all 241 cells were included and from one donor all 545 cells. In total, 6786 cells were acquired following quality control (QC) and integrated as shown in Figure 3A.

Melanocytes, fibroblasts, trabecular meshwork, Schwann cells, macrophages, T and B cell, smooth muscle, RPE, CECs, pericytes and mast cell clusters were present in the adult RPE–choroid sample similar to the fetal RPE–choroid tissues (Fig. 3A). A small fraction of rod photoreceptors was also identified potentially because of a small fragment of neural retina being pulled away with the RPE–choroid tissue. The pigmented cells were identified as melanocytes (cluster 0 and 3) and RPE cells (cluster 11) characterized by high expression of *MLANA* and *RPE65*, respectively. Fibroblasts (cluster 2) displayed high expression of *IGFBP5* and the closely situated pericytes (cluster 1) were distinguished by high expression of *ANGPT1* and *ACTN1.* Immune cells were characterized by high expression of CD69 in T cells (cluster 7), *CD79A* in B cells (cluster 15), *KIT* in mast cells (cluster 16) and CD68 and CD14 in macrophages (clusters 6 and 8). The CECs (cluster 12) were easily identified by the high expression of *VWF,* whereas smooth muscle cells (clusters 10 and 14) were defined on the basis of high expression of *ACTA2 and TAGLN.* Two clusters of Schwann cells (5 and 17) were identified by the high expression of *PLP1*: those were defined as myelinating (cluster 17) and non-myelinating (cluster 5) by the high expression of *MPZ* and *SCN7A*, respectively (Supplementary Material, Table S1, Fig. 3A).

Once all cell clusters were identified, the percentage of each cell type was compared between donors eyes of different ages (Fig. 3B). No consistent trends (increase or decrease in percentage as donor age increased) were observed except non-myelinating Schwann cells (cluster 5), which showed the highest percentage in the younger donor (51 years old) choroid. Nonmyelin forming Schwann cells, are thought to act as the ‘first responders’ in their neighbourhoods (46), hence a decrease in number, may suggest an inferior response of ageing choroid to injury or disease. These findings however need to be replicated in a larger number of donors at different ages to ensure that the trend we have observed is not because of sensitivity of these cells to single-cell dissociation, which may cause variability between donor choroids at different ages.

**Figure 4:**
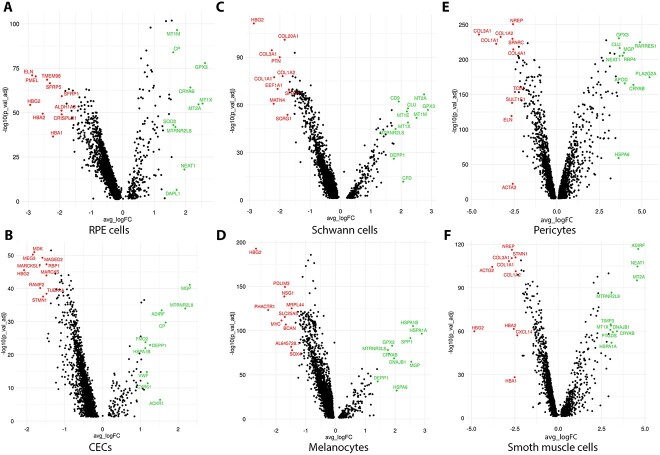
Volcano plots showing differentially expressed genes between fetal development and adult in RPE cells (**A**), CECs (**B**), Schwann cells (**C**), melanocytes (**D**), pericytes (**E**) and smooth muscle cells (**F**). Top 10 upregulated and downregulated genes are shown in green and red colour, respectively. An FDR threshold of <0.05 was applied.

To assess changes in gene expression profile between fetal and adult RPE–choroid tissues we performed DEG analysis (Supplementary Material, Table S5). Strikingly genes encoding proteins involved in oxidative stress response (47,48) and detoxification from heavy metals including *SOD2, GPX3, MT1M, MT1X, MT2A, CRYAB* and/or unfolded protein response (*HSPA1A, HSP1B*) were amongst the top 10–20 most upregulated genes in the adult RPE, CECs, Schwann cells, melanocytes, pericytes and smooth muscle cells (Fig. 4A–F), which could be explained by the fact that fetal cells *in utero* are not exposed to oxidative stress damage and are heavily protected by the maternal environment. Nonetheless, an upregulation of metallothionein genes was also reported when adult pericytes were compared with infant counterparts (43), indicating that the lower metallothionein gene expression in fetal pericytes may not be linked to cell exposure to oxidative stress damage. We also noted a significant increase in expression of some of the genes encoding proteins of HLA-class I (*HLA-A, HLA-B, HLA-C*) and HLA-class II (*HLA-DP, HLA-DRA, HLA-DQA2, HLA-DPA1*), suggesting an upregulation of antigen presentation and processing pathway in the adult RPE, CECs, Schwann cells, melanocytes and smooth muscle cells.

A recently published study (49) comparing the adult versus infant choroid has reported downregulation of genes encoding proteins involved in angiogenesis in the adult choriocapillaris as well as upregulation of numerous genes involved in the construction of extracellular matrix in the infant pericytes. Our DEG analyses support these findings, revealing upregulation of key angiogenesis (e.g. *ID1, ID4, ANGPT1, HES1, CD34, PDGFRA, PDGFRB, CD34, KDR*) and extracellular matrix genes (e.g. *COL1A1, COL3A1, COL1A2*) in the fetal CECs and pericytes, respectively, when compared with the adult equivalent cells (Supplementary Material, Table S5). Other key similarities to this published study include upregulation of genes encoding proteins involved in oxidative stress response (e.g. *MT1A, MT1X*) and antigen processing and presentation (e.g. *HLA-F, HLA-A, HLA-B, HLA-C, HLA-H, HLA-DQB1, HLA-DQA1*) in the adult CECs and pericytes. Such replicability of findings indicates that gene expression changes between the fetal and adult cell types identified in the RPE–choroidal tissue are reflective of underlying biological differences during fetal, postnatal and ageing process.

### Single-cell RNA-Seq of macular and peripheral AMD patient choroid–RPE samples

**Figure 5:**
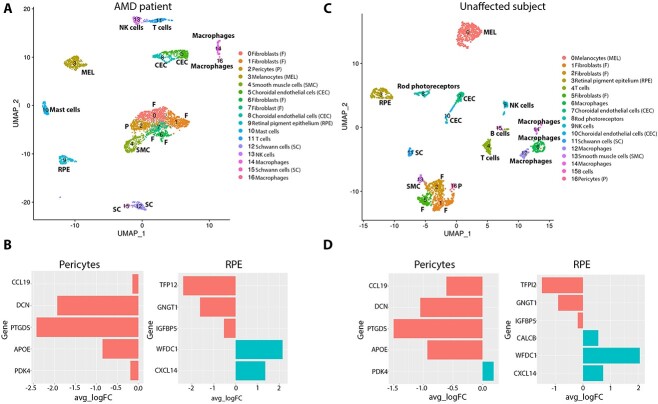
Single-cell RNA-Seq of human RPE–choroid tissues of an intermediate AMD patient and unaffected subject. (**A**) and (**C**) Integrated UMAP of macular and peripheral single-cell RNA-Seq of intermediated AMD patient (A) and unaffected subject (C) revealing the presence of 17 cell clusters; (**B**) and (**D**) Differential expression of macular versus peripheral pericytes, and RPE cells in the intermediate AMD patient (B) and unaffected subject (D). For each gene, the average log fold change is shown on the *x* axis. Genes enriched in the peripheral region and macular region are shown in red and green colour, respectively.

The macular and peripheral choroid–RPE tissues were dissected from a person diagnosed with intermediate AMD (Supplementary Material, Fig. S5) and an unaffected subject (Supplementary Material, Table S1) as shown in the Materials and Methods section and Supplementary Material, Figure S6. The macular and peripheral samples were dissociated to single cells and profiled by single-cell RNA-Seq (Supplementary Material, Table S1). Samples were profiled to a mean depth of 11 590 counts/cell and 3065 genes/cell. The samples from each subject were integrated by harmony batch correction. The cells from all four samples were downsampled to 1000 cells. In total 2000 cells were acquired from the AMD patient and unaffected subject each following quality control and integrated by harmony batch correction as shown in Figure 5A and C. Similar cell clusters to adult RPE–choroid tissue described above including RPE cells, CECs, melanocytes, Schwann cells, smooth muscle cells and pericytes were defined in both the AMD and unaffected subject macula-periphery integrated samples. To validate the macular and peripheral nature of the samples, the expression of region-specific markers defined by Voight *et al*. was assessed in the pericytes and RPE cell clusters (16). As expected, *CCL19, DCN, PTGD* and *APOE* were enriched in peripheral pericytes, whereas *PDK4* was enriched in the macular pericytes of the unaffected subject (Fig. 5D). A similar pattern was observed in pericytes of the AMD patient with the exception of *PDK4*, which was enriched in the peripheral region (Fig. 5B). *TFPI2, GNGT1* and *IGFBP5* were enriched in the periphery, whereas *WFDC1* and *CXCL14* were enriched in the macular RPE in both the AMD patient and unaffected control (Fig. 5B and D).

**Figure 6:**
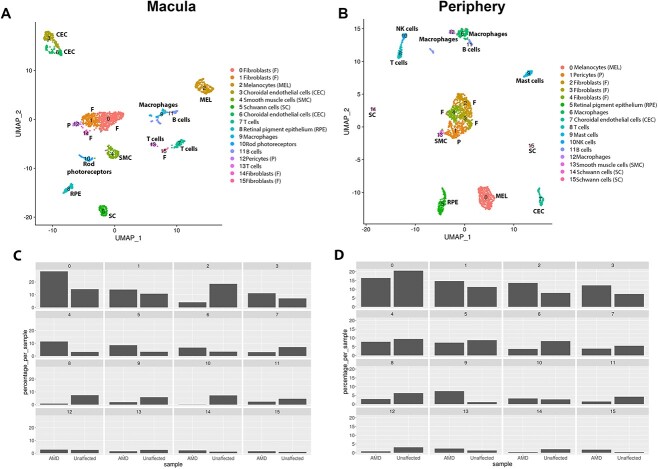
Comparative single-cell RNA-Seq of macular and peripheral region of the intermediate AMD patient versus unaffected subject. (**A**) and (**B**) Integrated UMAP of macular (A) and peripheral (B) regions showing the presence of 16 cell clusters. (**C**) and (**D**) Cell type representation in RPE–choroid tissue in the macular (C) and peripheral (D) region. The subject identity is shown on the *x* axis and cluster number on top of each panel.

Next, we went on to investigate changes in transcriptional signature and percentage between RPE cells, CECs, smooth muscle cells, melanocytes and Schwann cells between the AMD patient and unaffected subject in the macular and peripheral region (Fig. 6A and B). A reduction in the percentage of RPE cells (cluster 8) was observed in the macular region of the AMD patient when compared with the control (Fig. 6C); this was not the case for the peripheral region (cluster 5) (Fig. 6D). DEG analysis revealed cadherin 19 (*CDH19*) to be significantly upregulated in macular RPE cells of the AMD patient when compared with unaffected subject (Supplementary Material, Table S6). No changes in *CDH19* expression were observed when the peripheral RPE cells were compared between the AMD patient and the unaffected subject (data not shown). The cadherins are calcium-dependent cell adhesion proteins, involved in the structural and functional organization of cells in various tissues. The integrity of RPE cells is maintained in part by adherens junctions, which are composed of cadherin homodimers and p120-, β-, and α-catenins linking to actin filaments. Mutations in four cadherin, namely *CDHR1, CDH23, PCDH15, CDH3*, the latter being the dominant RPE cadherin, have been identified as causes of inherited retinal degeneration (50), including cone-rod dystrophy, macular dystrophy and Retinitis Pigmentosa. To date, there are no reports of CDH19 involvement in AMD, although a recent study has demonstrated an upregulation of many proteins involved in cell adhesion and extracellular matrix (ECM) regulation in geographic atrophy patient cohort (51). It thus remains to be investigated further if the *CDH19* overexpression in the macular RPE region of the AMD patient bears any functional significance on the loss or migration of RPE cells (52). Our comparative analyses of AMD and unaffected subject suggested also a reduction in the number of melanocytes, but only in the macular region of the intermediate AMD patient (Fig. 6C and D). Ingenuity Pathway Analysis (IPA) further supported loss of melanocytes from the AMD macula, showing cell death as the most upregulated molecular function in the melanocyte cluster (activation score 4.796).

**Figure 7:**
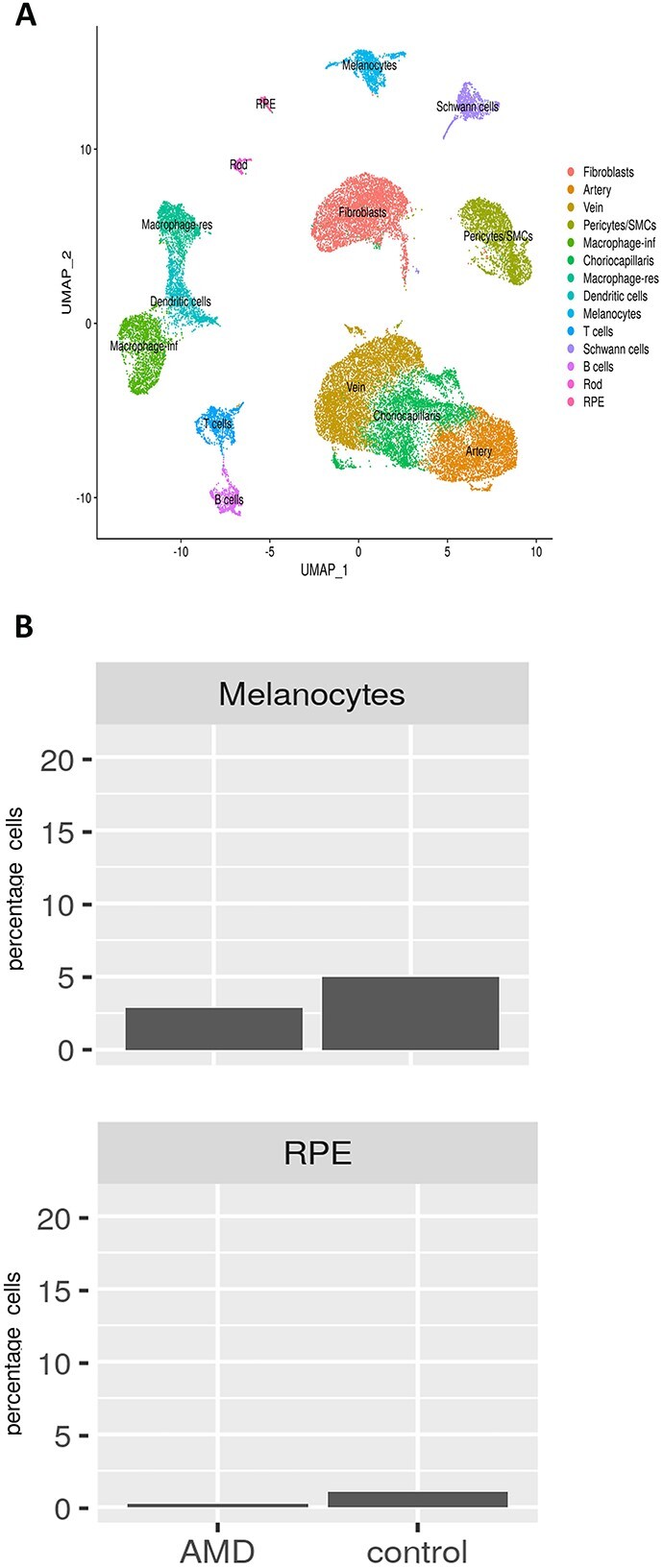
Comparative single-cell RNA-Seq of RPE–choroid macular regions obtained from 12 AMD patients [one intermediate AMD presented in Figure 6 + nine early atrophic AMD (53) + two neovascular AMD (60)] and 11 unaffected controls [one control presented in Figure 6 + 10 additional controls (60)]. (**A**) Integrated UMAP of macular regions showing the presence of various cell clusters. (**B**) Cell type representation in RPE–choroid tissue in the macular region. The subject identity is shown on the *x* axis and cluster number on top of each panel.

Since our data were derived from only one intermediate AMD donor and one unaffected subject, we integrated our single-cell RNA-Seq data of macular RPE–choroid tissue with macular choroids obtained from nine early atrophic, two neovascular AMD patients and 10 unaffected controls (53, Supplementary Material, Table S1). The data were clustered using the cell specific markers defined by Voight *et al*. recent publications (11, 60, Supplementary Material, Table S7). A side-by-side comparison of integrated data obtained from our intermediate and 11 AMD patients reported by Vought *et al.* (53) against our unaffected subject and 10 unaffected controls demonstrated a lower percentage of RPE cells and melanocytes in the macular AMD samples compared with the controls (Fig. 7A and B), corroborating our findings from a single patient versus single control analysis.

Loss of endothelial cells from the choriocapillaris is observed during ageing (53) and is one of the earliest detectable events in AMD (54–56). We did not see significant changes in CECs frequency between the AMD patient and unaffected subject either in the macular or peripheral region (Fig. 6C and D). However, the IPA revealed activation of cell death (Supplementary Material, Fig. S7) as one of the most enriched pathways when macular AMD CECs were compared with equivalent cells from the unaffected subject. Strikingly, IPA also revealed the activation of cell proliferation and cell migration concomitantly to cell death. Compensatory proliferation, a process initially identified in lower organisms during the regeneration of lost tissue (57,58), is an evolutionarily conserved process that also functions in mammals, its main function being repopulation of lost tissue. Intertwining of cell death and cell proliferation may explain the similar frequency of CECs in the macular region of intermediate AMD and unaffected subject. On the other hand, cell migration may underline the abnormal intravasation of choroid vessels into sub-RPE-BL (59), leading to wet AMD.

We observed significant overlaps in DEG lists between our study and that published recently by Voight *et al.* (60). for both upregulated (CECs 50%, Melanocytes 37%, Schwann cells 83%, pericytes 50% and SMCs 53%) and downregulated genes (CECs 38%, melanocytes 40%, Schwann cells 78%, pericytes 29%, SMCs 33%). One of the most differentially expressed upregulated gene in CECs of AMD patient identified in our study, the damage associated *SPARCL1* (average log2 fold change 0.47) was also enriched in the CECs of early atrophic AMD donors studied by Voigt *et al*. (average log2 fold change 0.47). Matrix Gla protein encoding gene (*MGP*) was also the highest differentially expressed and upregulated gene in melanocytes of AMD patients in both studies. Although our DEG lists contained less genes this could be because of the fact that only one AMD patient was compared with one unaffected subject in our study versus 12 AMD versus 10 controls in the Voigt *et al*. study. In addition, our DEG lists were filtered for *P* adjust < 0.05, whereas this or FDR filtering was not applied to the published comparative study (60).

## Discussion

Single-cell sequencing methods have provided exciting opportunities for understanding gene expression in tissues comprised of several cell types such as choroid. To date several single-cell transcriptome studies of human choroid have been performed in infants and adults demonstrating a pro-inflammatory gene expression, particularly in endothelial cells (49). In the context of early and neovascular AMD, these studies have revealed high expression of genes associated with CECs damage, demonstrating early degeneration of these cells during the course of AMD pathogenesis (60). RPE itself has been characterized at the single-cell level at both fetal and adult development as well as in the context of AMD pathogenesis (16,61). These studies have demonstrated an upregulation of genes related to extracellular structure organization, lipid biosynthesis and the canonical retinoid cycle during RPE development. Nonetheless at to the best of our knowledge, conjoined studies looking at changes in both RPE and choroid during the course of development, healthy adult and AMD have not been reported to date.

In this current study, we performed single-cell RNA-Seq of RPE and choroid dissected from four fetal samples covering the 12–21 PCW of development as well as five adult eyes, providing a comprehensive profiling of all the cell types present in these tissues and key transcriptional changes occurring during their development. Our data demonstrate that while some of these cell types are still in a proliferative state (e.g. Schwann cells), others are in the process of terminal differentiation (e.g. RPE, melanocytes, smooth muscle cells) or acquiring features (e.g. antigen presentation, immunomodulation) that will define their characteristics in the adult.

Oxidative stress and inflammation are key pathological events that are implicated in human AMD. Using an *in vitro* model, Kim *et al.* have shown that oxidative stress first affects retina in a persistent manner (62), with the RPE and choroid stress developing in a delayed and transient manner. These findings suggest a differential and tissue specific sensitivity to oxidative stress when the retina, RPE and choroid are compared. Our single-cell RNA-Seq analysis demonstrated a notable feature in all cell types examined (RPE cells, melanocytes, Schwann cells, pericytes, smooth muscle cells and CECs) within RPE and choroid tissues, this being a significant increase in expression of genes involved in oxidative stress response and detoxification from heavy metals, suggesting a better defence against oxidative stress in the adult RPE and choroid tissues. These findings are important for the field of regenerative medicine, as clinical trials are underway to replace lost or damaged RPE with pluripotent stem cell derived RPE cells (63), which transcriptionally mimic the fetal counterparts (64–66). Together these data suggest the need for reducing or preventing the generation of ROS coupled with transplantation of pluripotent stem cell derived RPE cells, especially for diseases such as AMD where oxidative stress is a key hallmark of disease pathogenesis.

Previous studies in the field have compared gene expression at the single-cell level between AMD patients and unaffected subjects (16,60). However, in all these studies, the RPE and choroid tissues were obtained from atrophic AMD patients. We took the opportunity to analyze the RPE and choroid tissue from a patient with intermediate AMD to better understand the sequence of cell loss and degeneration during AMD progression. Our findings suggest a macular specific loss of RPE cells and melanocytes, corroborating published reports of RPE cell death and migration accompanied by the release of organelle clusters (52), and RPE atrophy in the macula of AMD patients (67,68). It is unlikely that the under-representation of melanocytes in the macular region represent a technical issue (e.g. loss of cells during cell dissociation or capture) for this cluster was fully represented in the peripheral choroid. Published evidence indicates that RPE cells overly soft drusen material, which is only found in the macula. This could indicate that macular RPE cells may be less attached to the underlying Bruch’s membrane, although the very same study has shown full RPE coverage over soft drusen deposits (69). It is possible that the RPE cells and melanocytes in the macular region of AMD patients are more fragile than their peripheral counterparts and thus could be lost during the single-cell dissociation procedures. A recent study, which used cutting-edge imaging and analysis methods, showed loss of RPE cells in the fovea and most of the centre part of the parafoveal region in unprocessed macular samples of AMD patients (70), which indicates that the reduced fraction of RPE cells we have unveiled from the single-cell RNA-Seq analysis may not be because of the cell dissociation procedure, but perhaps reflect a real and specific finding characterizing the intermediate AMD stage of the disease. This being said, one has to be careful of not underscoring potential ‘RPE’ removal together with the retina during the dissection process in specific samples, which may lead to RPE cell under-representation in some samples. To validate our findings, we integrated our data with those obtained from single-cell RNA-Seq profiling of macular choroid of an additional 11 early AMD patients and 10 unaffected controls (53), and showed a similar reduction in RPE cells and melanocytes in the macular region of AMD patients. This notwithstanding the samples used by Voight *et al*. (60) were preselected for expression of CD31, hence the numbers of RPE cells and melanocytes that ‘escape’ selection is not a good indicator of how many of these were present initially. Together these findings suggest that a difference in macular RPE/melanocyte numbers may be present in AMD; but given the complex processing of tissues required for single-cell RNA-Seq that is prone to technical artefacts, these findings need to be validated by additional techniques (e.g. *in situ* microscopy, spatial transcriptomics, flow cytometry and/or morphometric findings) in a larger number of AMD patients and controls.

The pathway enrichment analyses demonstrated the onset of cell death programme in CECs, corroborating previous reports showing an increase in expression of genes (such as *RGCC* and *SPARCL1*) that induce or respond to apoptosis in endothelial cells of AMD patients (16,60). Intriguingly, the activation of cell death was closely intertwined with cell proliferation and cell migration activation, the latter being a key event in angiogenesis and choroid neovascularization in AMD. The loss of CECs in AMD leads to loss of vascular support to the RPE, which in turn releases angiogenic signals which stimulate the abnormal intravasation of choroid vessels into the subretinal layers. From our data, it appears that both the programme of cell death and cell migration are present in macular CECs of the intermediate AMD patient, despite the apparent maintenance of CECs number. It would be interesting to follow this process and define the point of CECs loss and/or migration during progression of AMD from the intermediate to the late stages, but this is heavily dependent on sample availability at the right stage of disease progression.

There are several limitations to this study. First only four fetal samples were processed by single-cell RNA-Seq and thus it would be desirable to include a larger number of samples covering a more extensive window of fetal development from the earliest available stages (e.g. 7 PCW) until 21–22 PCW. In addition, the sample collection post-mortem time was different between each sample, which could lead to retention of ribosomal RNA; however we did not see large variances between the samples with regard to ribosomal content, except the macular and peripheral samples taken from the intermediate AMD patient, which were processed within 1.5 h post eye retrieval and showed the lowest ribosomal content. Given the scarcity of RPE and choroid tissues from patients with intermediate AMD, we were unable to perform validation studies at the protein level.

In summary our data provide a rich resource for the community to investigate gene expression within each of these cell types present in the RPE–choroid tissue during development as well as the healthy adult and AMD disease setting. The raw and processed data from this analysis has been deposited to gene expression omnibus (accession number GSE210543) to enable other researchers in the field to use and expand on these data and to gain new insights that would facilitate the treatment of AMD.

## Materials and Methods

### Human tissue donation

Adult and fetal human donor eyes were donated for research following informed consent by family members and in accordance with the Declaration of Helsinki. All adult human tissue was provided by NHS Blood and Transplant Tissue and Eye Services, and the Newcastle and Sunderland NHS Trust following ethical approval (18/YH/04/20). The human fetal material was provided by the Joint MRC/Wellcome Trust (MR/R006237/1) Human Developmental Biology Resource (71) under ethics permission 08/H0906/21 + 5 issued by the NorthEast-Newcastle and North Tyneside 1 Research Ethics Committee. All donor information is reported in Supplementary Material, Table S1**.** RPE and choroid tissues from whole eyecups of five adult donor tissues and four fetal samples from 12 to 21 PCW terminations were used for scRNA-Seq. The eye globes were dissected in Hank's Balanced Salt Solution (HBSS) immediately after collecting tissue. Two further eye samples from one person with intermediate AMD and one unaffected subject were obtained through exenteration procedures for advanced facial cancers performed at Royal Victoria Infirmary (RVI), Newcastle (Supplementary Material, Table S1). To acquire central and peripheral RPE and choroid from these, a 7 mm macular-centred punch and a 7 mm peripheral trephine from the inferotemporal region were used, as shown in Supplementary Material, Figure S6. The neural retina was separated from the underlying RPE and choroid.

### Single cell RNA-Seq

The excised RPE and choroid tissues were dissociated to single cells using a multi tissue dissociation kit (Miltenyi Biotec). Dissociation time varied from 15 to 45 min depending on the age of the tissue and a gentleMACS Dissociator (Miltenyi Biotec) was used to aid dissociation of the adult tissue. Cell count and viability were monitored using a Tali Image-Based Cytometer and Viability Kit (Thermo Fisher Scientific). For scRNA-Seq, cells were captured, and libraries generated using the Chromium Single Cell 3′ Library & Gel Bead Kit, version 3 (10× Genomics). scRNA-Seq libraries were sequenced to 50 000 reads per cell on an Illumina NovaSeq 6000.

CellRanger version 3.01 was used to de-multiplex samples, align to the human genome (GRCh38) and create gene expression matrix. Quality of the samples was assessed and any cells with fewer than 1000 reads, 500 genes or 20% mitochondrial reads were removed before further analysis (Supplementary Material, Fig. S8). DoubleFinder (version 2.0.3) was used to find and remove any doublets. Each sample was log normalized using Seurat (version 3.2.3). The top 2000 highly variable genes and PCA dimension reduction were applied to the data. The samples were integrated using Harmony batch correction.

The integrated datasets were clustered using a resolution of 0.6. Marker genes were identified using the Seurat FindMarkers function. Using these marker genes, clusters were then assigned cell types and visualized using UMAP. Differentially expressed genes were generated using the FindMarkers function. A core analysis from Qiagen IPA was used to investigate pathways associated with the differentially expressed genes.

*Conflict of Interest statement*. The authors declare no conflict of interest.

## Supplementary Material

Figure_S1_ddad007

Figure_S2_ddad007

Figure_S3_ddad007

Figure_S4_ddad007

Figure_S5_ddad007

Figure_S6_ddad007

Figure_S7_ddad007

Figure_S8_ddad007

Table_S1_ddad007

Table_S2_ddad007

Table_S3_ddad007

Table_S4_ddad007

Table_S5_ddad007

Table_S6_ddad007

Supplementary_information_final_for_submission_ddad007

## Data Availability

The single cell RNA-Seq data datasets produced in this study are deposited in the Gene Expression Omnibus (GSE210543).
