# Supplementary figures and images for: Single-cell RNA sequencing reveals transcriptional changes of human choroidal and retinal pigment epithelium cells during fetal development, in healthy adult and intermediate age-related macular degeneration
Source: Hum Mol Genet. 2023 Jan 16;32(10):1698–710. doi: 10.1093/hmg/ddad007 (PMC10162434; doi:10.1093/hmg/ddad007)

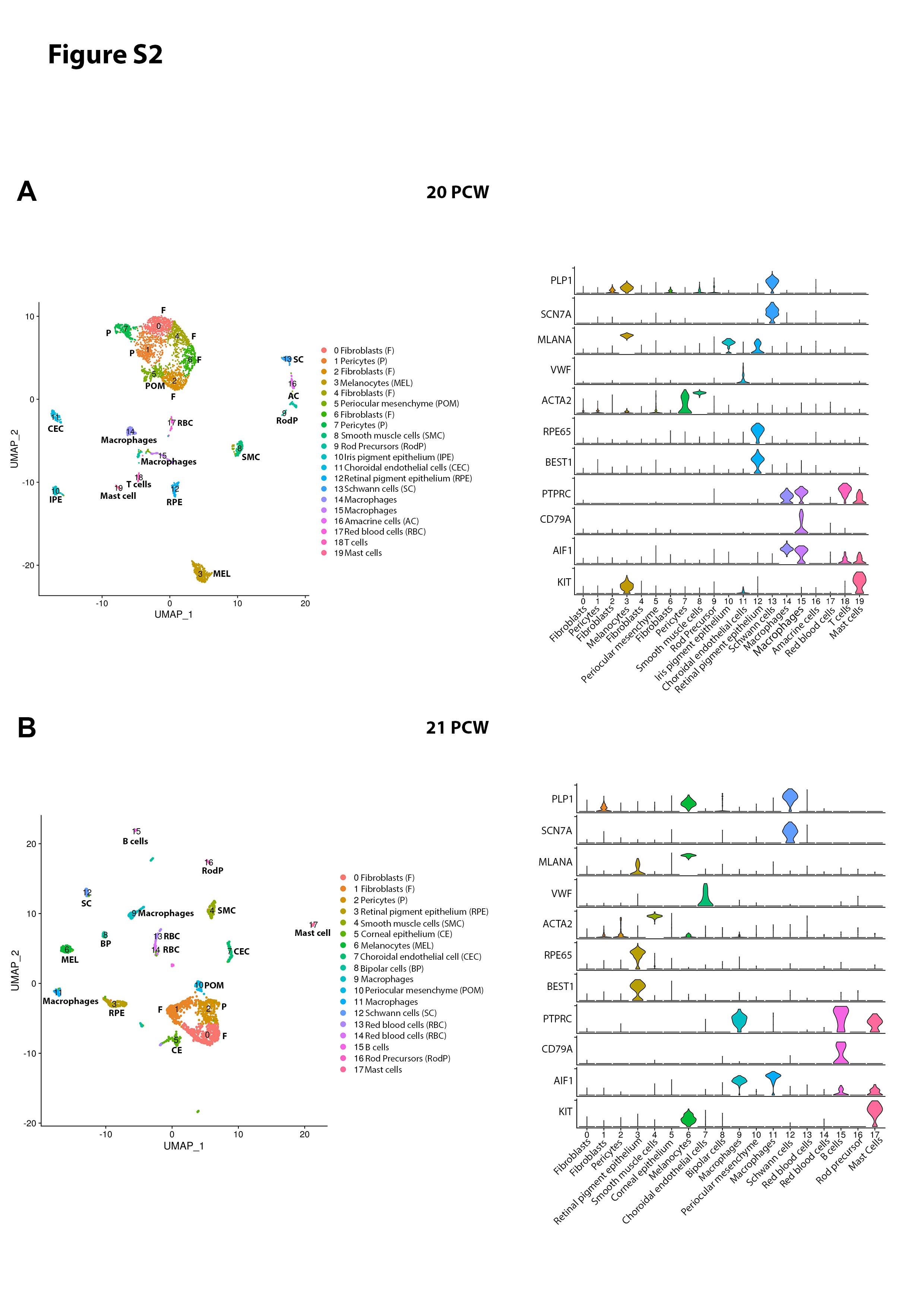

Supplement: Figure_S2_ddad007 [file figure_s2_ddad007.jpeg]

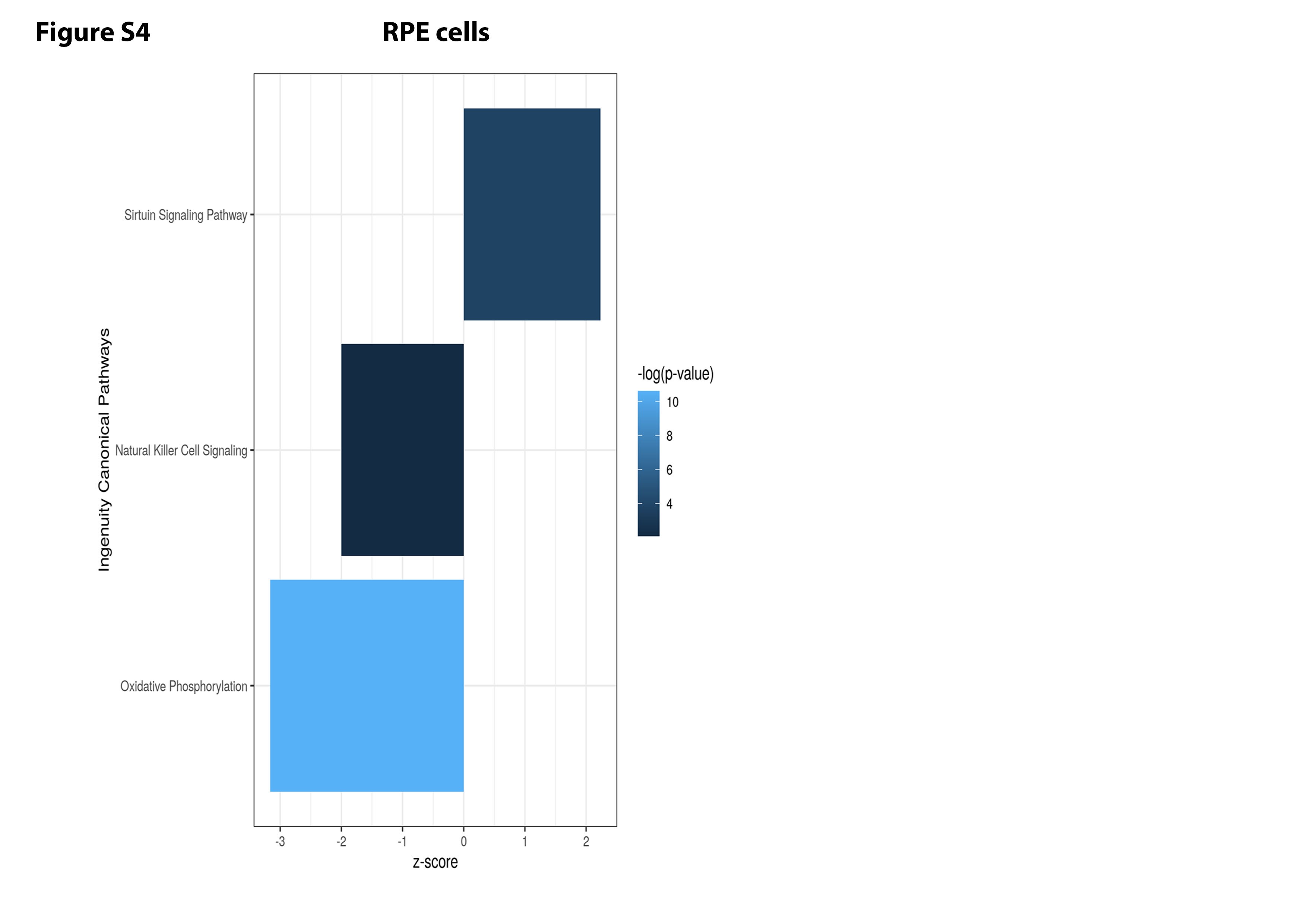

Supplement: Figure_S4_ddad007 [file figure_s4_ddad007.jpeg]

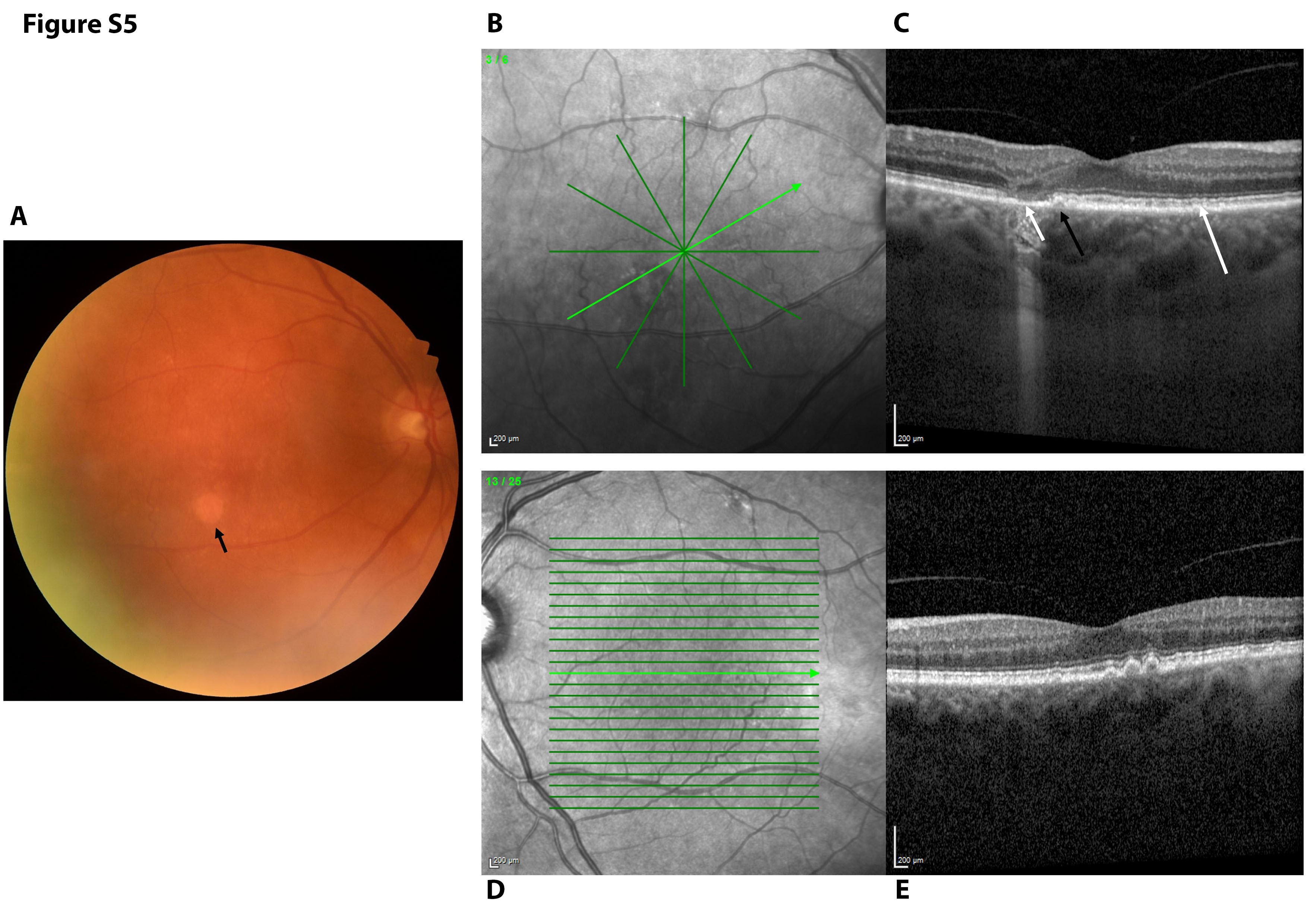

Supplement: Figure_S5_ddad007 [file figure_s5_ddad007.jpeg]

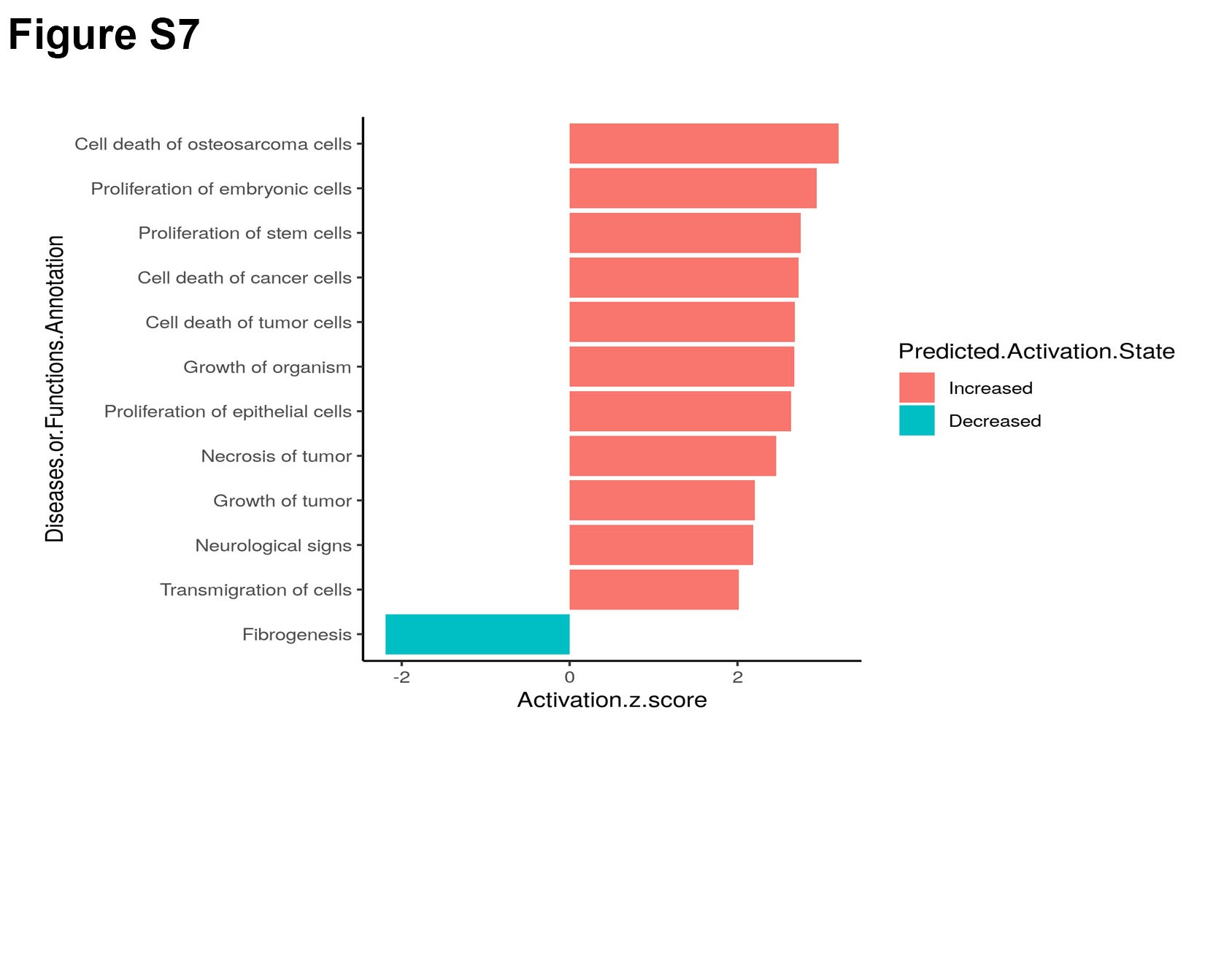

Supplement: Figure_S7_ddad007 [file figure_s7_ddad007.jpeg]

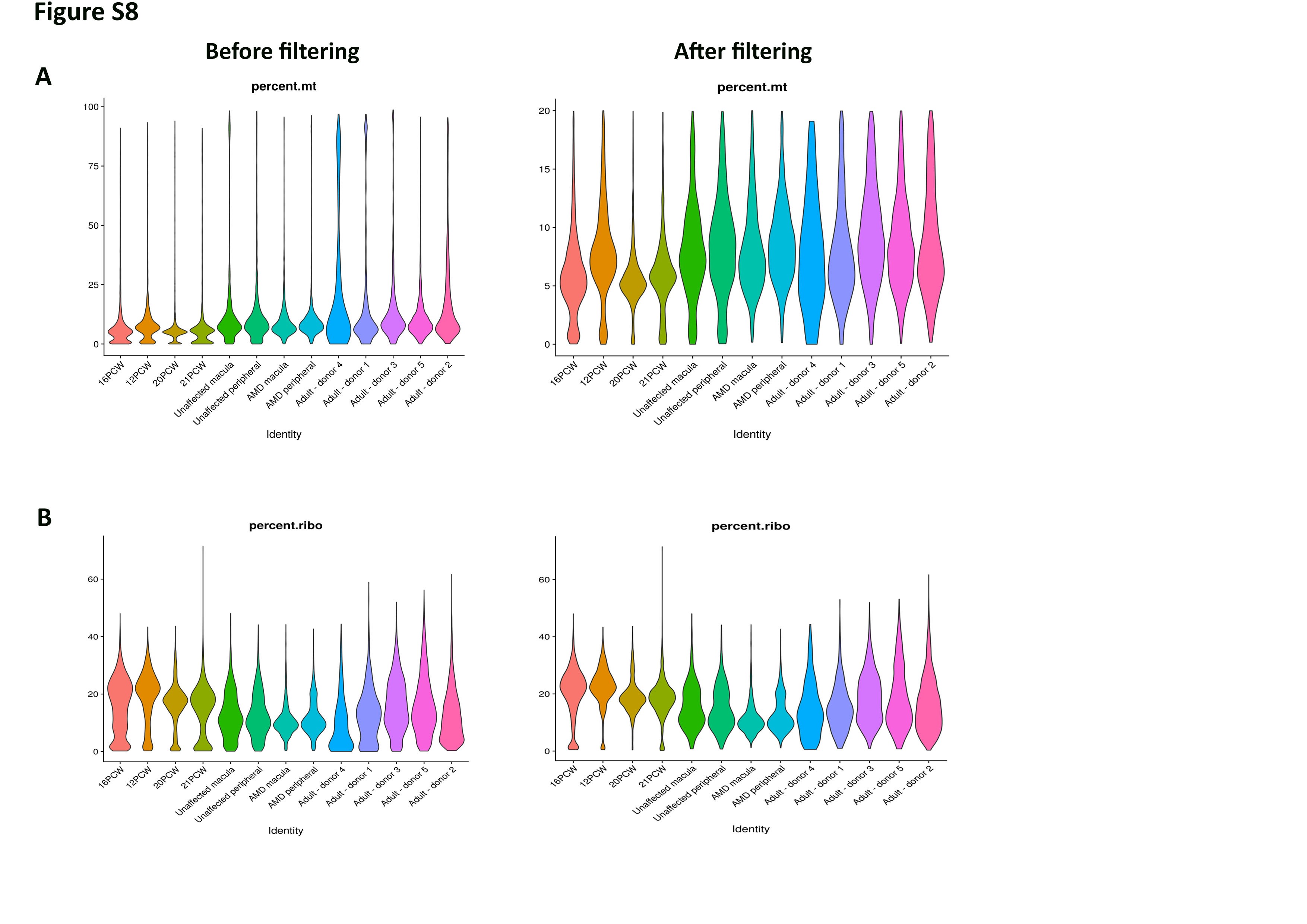

Supplement: Figure_S8_ddad007 [file figure_s8_ddad007.jpeg]
