# Supplementary material for: Single-cell RNA sequencing reveals transcriptional changes of human choroidal and retinal pigment epithelium cells during fetal development, in healthy adult and intermediate age-related macular degeneration
Source: Hum Mol Genet. 2023 Jan 16;32(10):1698–710. doi: 10.1093/hmg/ddad007 (PMC10162434; doi:10.1093/hmg/ddad007)
Supplement: Supplementary file 2 [file supplementary_information_final_for_submission_ddad007.zip › Supplementary_information_final_for_submission_ddad007.odt]

Supplementary information

Figure S1: Single cell RNA-Seq of fetal human RPE - choroid tissue. A) UMAP (left) revealing the presence of 15 cell clusters in the 12 PCW choroid - RPE tissue and violin plots (right) showing the expression of key cell type specific markers; B) UMAP (left) revealing the presence of 22 cell clusters in the 16 PCW choroid - RPE tissue and violin plots (right) showing the expression of key cell type specific markers.

Figure S2: Single cell RNA-Seq of fetal human RPE - choroid tissue. A) UMAP (left) revealing the presence of 20 cell clusters in the 20 PCW choroid - RPE tissue and violin plots (right) showing the expression of key cell type specific markers; B) UMAP (left) revealing the presence of 18 cell clusters in the 21 PCW RPE - choroid tissue and violin plots (right) showing the expression of key cell type specific markers.

Figure S3: Violin plots showing gradual changes in gene expression with age in RPE cells (A), CECs (B) and melanocytes (C) during human fetal retinal development.

Figure S4: Pathway enrichment analysis of differentially expressed genes between the early and late fetal RPE cells. Differentially expressed genes between early and late fetal development were generated for RPE cells using the Seurat FindMarkers function. IPA Pathway Analysis was used to predict canonical pathways, using a z score with an absolute value of 2 as cut off.

Figure S5: Clinical photographs showing the retinal and RPE abnormalities in the intermediate AMD patient. A) Colour fundal photograph of exenterated eye showing fine drusen with an area of geographic atrophy  inferotemporal to the foveal centre (black arrow). Image hazy secondary to corneal opacity due to exposure keratitis secondary to lid disease; B, C) Corresponding spectral domain optical coherence tomography (SDOCT) scan through area of GA (short white arrow), showing presence of both typical sub RPE drusen (black arrow) and subretinal drusenoid deposits (long white arrow); D, E) Fellow eye SDOCT showing similar features (taken with higher scan averaging and hence improved resolution). (vitreomacular adhesion present in both eyes). AMD staging based on the AREDS grading system (72).

Figure S6: Schematic presentation showing the dissection of macular and peripheral RPE-choroid regions from the unaffected subject. 

Figure S7: Pathway enrichment analysis of differentially expressed genes between the macular CECs of intermediate AMD and unaffected subject. Differentially expressed genes were generated for RPE cells using the Seurat FindMarkers function. IPA Pathway Analysis was used to predict canonical pathways, using a z score with an absolute value of 2 as cut off.

Figure S8: Quality control showing removal of cells with  more than 20% mitochondrial reads (A). No filtering based on ribosomal content was performed (B).

Table S1: Single cell RNA-Seq delineating the expression of highly and differentially expressed genes for each cell cluster in all samples reported in this study.

Table S2: Differentially expressed genes (p< 0.05) between late and early fetal development in RPE cells, CECs, Schwann cells, melanocytes, pericytes and smooth muscle cells. Upregulated genes shown in green and downregulated genes in red shade.

Table S3: List of significant regulators of gene expression identified by IPA in late versus early fetal development in RPE cells, CECs, Schwann cells, melanocytes, pericytes and smooth muscle cells.

Table S4: List of IPA enriched pathways in late versus early fetal development in RPE cells, CECs, Schwann cells, melanocytes, pericytes and smooth muscle cells. NaN- z score could not be calculated.

Table S5: Differentially expressed genes (p< 0.05) between adult and fetal development in RPE cells, CECs, Schwann cells, melanocytes, pericytes and smooth muscle cells. Upregulated genes shown in green and downregulated genes in red shade.

Table S6: Differentially expressed genes (p< 0.05) between the intermediate AMD patient and unaffected subject in macular RPE cells, CECs, Schwann cells, melanocytes, pericytes and smooth muscle cells. Upregulated genes shown in green and downregulated genes in red shade.

Table S7: Integrated single cell RNA-Seq of the intermediate AMD patient and unaffected control reported in this study together with the single cell data of an additional 11 AMD patients and 10 unaffected controls published by Voight et al (53) delineating the expression of highly and differentially expressed genes for each cell cluster.
